# Perception of Male Caller Identity in Koalas (*Phascolarctos cinereus*): Acoustic Analysis and Playback Experiments

**DOI:** 10.1371/journal.pone.0020329

**Published:** 2011-05-25

**Authors:** Benjamin D. Charlton, William A. H. Ellis, Allan J. McKinnon, Jacqui Brumm, Karen Nilsson, W. Tecumseh Fitch

**Affiliations:** 1 Department of Cognitive Biology, University of Vienna, Vienna, Austria; 2 Koala Ecology Group, School of Biological Sciences, University of Queensland, Brisbane, Queensland, Australia; 3 Department of Environment and Resource Management, Moggill Koala Hospital, Bellbowrie, Queensland, Australia; 4 Lone Pine Koala Sanctuary, Brisbane, Queensland, Australia; University of Saint-Etienne, France

## Abstract

The ability to signal individual identity using vocal signals and distinguish between conspecifics based on vocal cues is important in several mammal species. Furthermore, it can be important for receivers to differentiate between callers in reproductive contexts. In this study, we used acoustic analyses to determine whether male koala bellows are individually distinctive and to investigate the relative importance of different acoustic features for coding individuality. We then used a habituation-discrimination paradigm to investigate whether koalas discriminate between the bellow vocalisations of different male callers. Our results show that male koala bellows are highly individualized, and indicate that cues related to vocal tract filtering contribute the most to vocal identity. In addition, we found that male and female koalas habituated to the bellows of a specific male showed a significant dishabituation when they were presented with bellows from a novel male. The significant reduction in behavioural response to a final rehabituation playback shows this was not a chance rebound in response levels. Our findings indicate that male koala bellows are highly individually distinctive and that the identity of male callers is functionally relevant to male and female koalas during the breeding season. We go on to discuss the biological relevance of signalling identity in this species' sexual communication and the potential practical implications of our findings for acoustic monitoring of male population levels.

## Introduction

Individually distinctive vocal signals and the ability to discriminate between individuals based on their vocalizations are documented in several mammal species ([1]–[3], for review see [4]). Indeed, we would expect signallers to be individually distinctive and receivers to differentiate between individuals where it is adaptive for them to do so [5]. For example, in social mammals individually distinctive vocalizations may be important for facilitating group cohesion [6], [7] recognizing kin [8] and warning group members of potential danger [9], [10]. In addition, mother offspring vocal recognition can be crucial for dependant young to survive [11]–[13]. However, vocal recognition and the ability to signal individual identity may also be important in inter and intra-sexual contexts. For instance, individually distinctive calls are important for delineating territories [14], [15] and the ability to distinguish between unfamiliar and familiar rivals could help prevent unnecessary contests between males [16]. In addition, over the course of a breeding season, females may become familiar with and prefer the vocalisations of certain males that can afford higher energy courtship displays [17]–[19].

Koalas (*Phascolarctos cinereus*) are solitary, arboreal mammals that are largely nocturnal [20]–[22]. Consequently, visual communication is likely to be restricted to infrequent associations, and koala vocal signals could be crucial for coordinating mating activities. The male koala's most conspicuous vocalisation is a low-pitched ‘bellow’ that is characterised by a continuous series of inhalations and short belch-like' exhalations [20], [23], and is particularly prominent during the annual breeding season [21], [22]. Although females are capable of bellowing they do so only rarely [23]. Moreover, a strong correspondence between male bellowing and breeding activity suggests that these calls play an active role during intra-sexual competition, and may even be used to attract females directly [22]. However, despite speculation about the possible functions of male koala bellows, it is not known whether these calls encode information about the caller's phenotype of potential importance in reproductive contexts.

The application of the source-filter theory [24] of vocal production to mammal calls allows us to make predictions about which acoustic characteristics have the potential to provide receivers with reliable information about the caller [4], [25], [26]. The source-filter theory states that mammal vocal signals are generated by the conversion of airflow from the lungs to acoustic energy by the larynx, the source, which is then filtered by the vocal tract. The rate the vocal folds in the larynx open and close determines the fundamental frequency (F0) of the vocalization and the supra-laryngeal vocal tract acts as a spectral filter, selectively transmitting certain frequencies termed vocal tract resonances or ‘formants’ [27]. Since inter-individual differences in laryngeal and vocal tract morphology are likely, both source and filter-related acoustic characteristics of male koala bellows have the potential to yield information on the caller's identity. In addition, the very low F0 of male bellows should highlight an individual's distinctive formant pattern, making these calls particularly well suited for identity cueing (as discussed by [28]).

Furthermore, although male bellowing occurs in response to other male bellows and after agonistic interactions or mating attempts [23], ‘spontaneous’ bellows often occur just after males have moved to a new location [20]. Although we cannot rule out the stimulating affects of olfactory signals, this may indicate that is important for males to advertise their presence to neighbouring conspecifics, and perhaps a more general broadcasting function for this call. In addition, koalas occupy overlapping ranges and roam more widely during the breeding season [22], [29]. Consequently, they will interact with others in adjacent territories more at this time and could familiarize themselves with the bellows of certain individuals. The perception of caller identity might prove adaptive in this species, allowing female koalas to exhibit mating preferences based on familiarity and male koalas to avoid known rivals that represent a threat to them. For this to occur, however, male koalas need to possess individually distinctive bellows, and male and female koalas need to distinguish between the calls of different males.

Here we use acoustic analyses based on source-filter theory to determine whether male koala bellows are individually distinctive vocalizations, and to assess the relative importance of different source- and filter-related acoustic features for coding individuality. We then use a habituation-discrimination paradigm [3], [30]–[34] to investigate whether male and female koalas can perceive differences between male callers. We predict that after habituating to a series of male bellows, both sexes will show a renewal of response levels to a bellow from a novel male. Such perceptual abilities could be adaptive in the contexts of intra-sexual competition and mate choice, and would raise further questions about the possible functions of identity cueing in koala sexual communication.

## Results

### Description of male bellows

**Figure 1:**
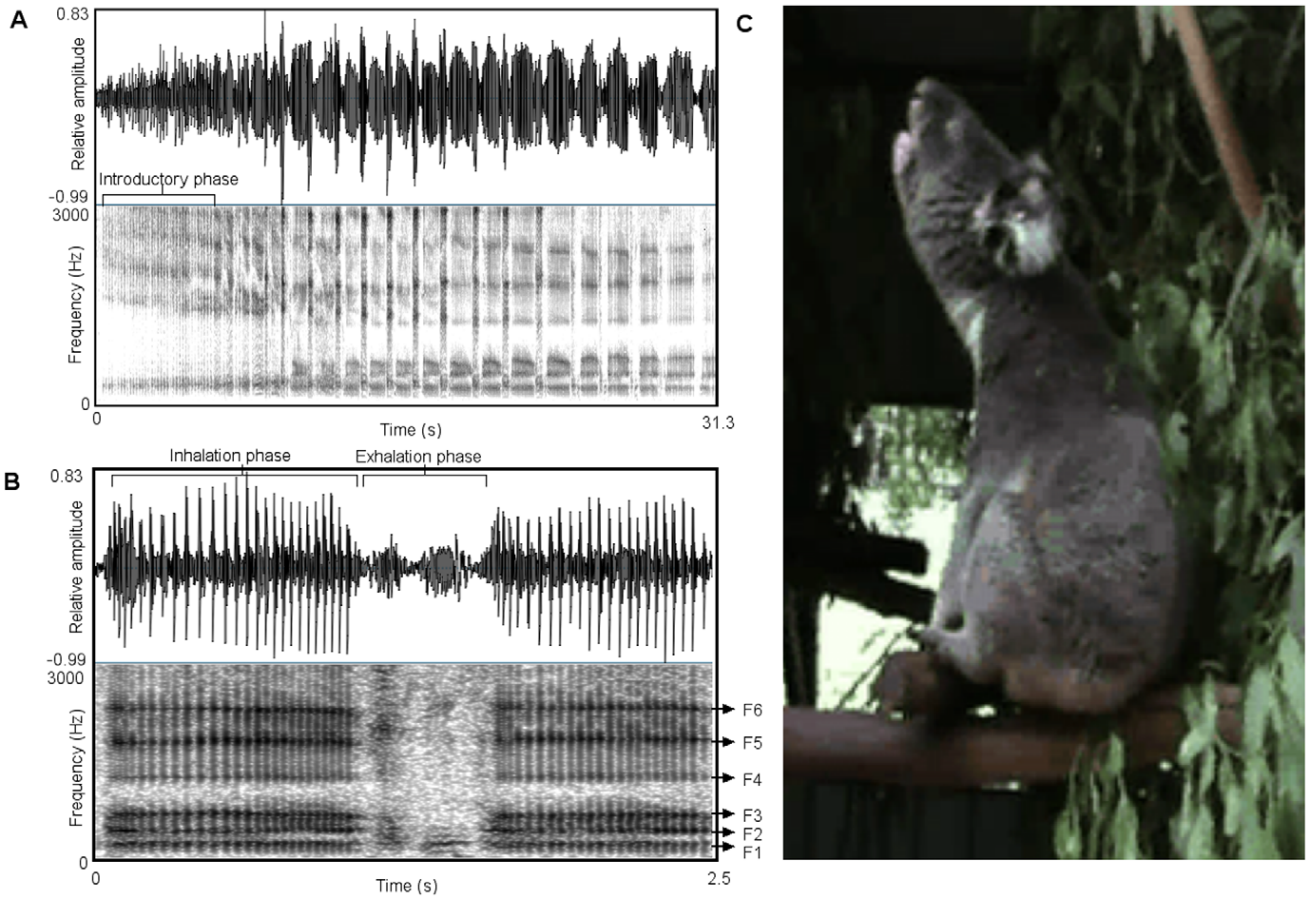
The left top panel (a) shows a spectrogram of a male bellow (spectrogram settings: FFT method; window length 0.05 s; time step = 0.004 s; frequency step = 10 Hz; Gaussian window shape; dynamic range = 35 dB). Male bellows are characterised by a ‘staccato’ introductory phase that is followed by a continuous series of inhalations and shorter ‘belch-like’ exhalations. The left lower panel (b) illustrates the pulse train structure and clear spectral prominences (labelled F1–F6) of the inhalation phases. The panel on the right (c) shows the typical posture adopted by a bellowing male koala.

Male koala bellows typically begin with a ‘staccato’ introductory phase that consists of abrupt amplitude onsets and offsets and no clear harmonic structure. This is followed by a continuous series of inhalations and shorter ‘belch-like’ exhalations (see Figure 1a and Movie S1). The inhalation phases of male bellows have a very low F0 (circa 25 Hz), making a pulse-train structure visible in the spectrograms (see Figure 1b). The pulses presumably represent glottal closure and the rate that they occur per second determines the F0 of these sections of the vocalisation. In addition, clear spectral prominences are present during the inhalation, and initial exhalation, phases of bellows that are likely to represent supra-laryngeal resonances (see Figure 1b). We only considered the inhalation sections of male bellows with a clear pulse-train structure and stable spectral prominences for the analysis of source- and filter-related features. Descriptive statistics for all the acoustic measures are given in Table 1.

**Table 1:** Descriptive statistics (*N* = 20) and tests of equality of group means between individuals for each of the acoustic measures used in the DFA.

Acoustic measures	*M*	s.d.	Minimum	Maximum	Wilks' lambda	*F_19,256_*	*P*
Duration	38.51	12.23	20.57	70.14	0.712	5.438	<0.001
Mean F0	27.07	5.77	18.95	40.69	0.610	8.613	<0.001
Maximum F0	61.45	12.18	32.80	82.80	0.663	6.848	0.085
Minimum F0	9.80	0.76	8.76	11.54	0.900	1.500	<0.001
Jitter	5.75	0.50	4.94	6.54	0.794	3.499	<0.001
Ampvar	14.11	1.89	10.82	17.30	0.531	11.908	<0.001
F1	216.94	17.85	186.27	247.57	0.539	11.505	<0.001
F2	416.68	28.92	362.50	465.13	0.337	26.557	<0.001
F3	660.79	55.26	560.00	772.67	0.390	21.050	<0.001
F4	1155.96	134.51	922.10	1360.25	0.117	101.622	<0.001
F5	1618.40	105.97	1427.27	1812.13	0.209	51.123	<0.001
F6	2131.99	143.23	1845.87	2473.20	0.270	36.513	<0.001
ΔF	355.81	23.12	312.16	405.29	0.139	83.131	<0.001

See text for definition of variables.

### Inter-individual differences in male vocal characteristics

A discriminant functions analysis (DFA) correctly classified 87.7% of 276 bellows to the 20 males, falling to 79.0% when the more conservative leave-one-out cross validation was applied. Compared to that expected by chance the level of classification was statistically significant for each individual and across all individuals (see Table 2: all P<0.001). In addition, the univariate analysis showed that all the acoustic features measured except minimum F0 differed significantly between individuals (see Table 1). Furthermore, 81.3% of bellows recorded on the last day for each individual were correctly classified using models trained with calls recorded on the other previous days, confirming that vocal individuality was stable across different days. The examination of the structure matrix shows that the main contributors to individual vocal distinctiveness were filter-related acoustic features, in particular the upper spectral prominences (F4–F6) and ΔF (see Table 3). In contrast, source-related features mainly loaded onto discriminant functions 3&4 that together only explained 16.8% of the variance and hence, were far poorer at classifying male bellows to individuals (see Table 3).

**Table 2:** Observed percentage (%) of correct classification against expected levels as calculated from group sizes.

Subject	*N*	Expected (%)	Observed (%)
Andy	18	6.5	94.4
Bagel	18	6.5	77.8
Bogie	11	4.0	90.9
Bunker	14	5.1	78.6
Denzel	15	5.4	100.0
Fitzroy	11	4.0	81.8
Kakadu	13	4.7	84.6
Maximus	14	5.1	85.7
Monacle	15	5.4	100.0
Mr Peabody	11	4.0	90.9
Neon	12	4.3	83.3
Orion	16	5.8	81.2
Otto	20	7.2	100.0
Patch	13	4.7	76.9
Squid	13	4.7	69.2
Sumo	13	4.7	84.6
TC	18	6.5	88.9
Wendell	11	4.0	81.8
Yabbie	10	3.6	100.0
Zagget	10	3.6	100.0
Mean	13.8	5	87.7

*P* values obtained using the Chi square statistic are all <0.001.

**Table 3:** DFA structure matrix showing pooled within-groups correlations between discriminating variables and the first four standardized canonical discriminant functions with eigenvalues >1 (explaining 85.7% of the variance).

Acoustic measures	Discriminant functions			
	1	2	3	4
F4	**0.826**	**−0.365**	−0.175	0.018
ΔF	**0.726**	**0.441**	−0.005	−**0.355**
F6	**0.364**	**0.530**	**0.358**	−**0.450**
F5	**0.515**	**0.307**	−**0.545**	−0.241
F3	0.276	0.194	−0.047	0.297
F1	0.162	−0.024	−0.066	−0.100
F2	**0.383**	0.130	−0.201	0.110
Ampvar	0.048	0.247	0.258	**0.537**
Mean F0	−0.083	0.062	−**0.346**	0.284
Maximum F0	0.024	0.149	−0.193	**0.370**
Duration	0.086	0.001	0.178	−0.135
Minimum F0	−0.038	−0.029	−0.041	−0.086
Jitter	0.015	0.057	0.187	0.192
Eigenvalue	10.3	2.6	2.0	1.2
% of Variance	55.0	13.8	10.7	6.1
Cumulative %	55.0	68.8	79.6	85.7

Discriminating variables are ordered by absolute size of correlation within function. Correlation coefficients >0.3 are in bold.

### Discrimination of caller identity

**Figure 2:**
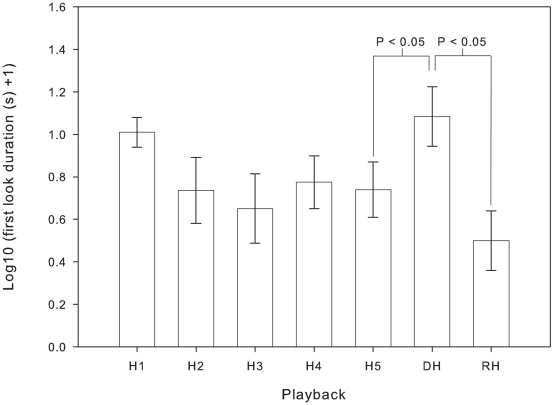
Mean ± SE of looking responses to the habituation-discrimination playback sequences.

A general linear model showed that the first look duration of subjects was significantly affected by playback type (*F*_1,10_ = 6.205, *P* = 0.008). Pairwise comparisons indicated a significant increase in first look duration between the last playback of the habituation phase (H5 = 7.50±2.40 s) and the dishabituation playback (DH = 17.22±3.85 s) (*P* = 0.016) (see Figure 2). In addition, a significant reduction in response duration between the dishabituation playback (DH = 17.22±3.85 s) and the rehabituation playback (RH = 5.01±2.48 s) was observed (*P* = 0.016), with the response levels falling back below that of the last habituation playback (H5) (see Figure 2). No sex differences in overall looking response were detected (*F*_1,10_ = 0.008, *P* = 0.931). In addition, the interaction effects indicated that male and female koalas were equally likely to dishabituate to the change in male caller (*F*_1,10_ = 0.398, *P* = 0.542) and rehabituate when re-presented with the male caller from the habituation phase (*F*_1,10_ = 1.313, *P* = 0.278).

## Discussion

### Inter-individual differences in male vocal characteristics

The DFA confirmed that male koala bellows are highly individually distinctive, with 87% of calls correctly assigned to individual callers (falling to 79% when the more conservative leave-one-out, cross validation approach was used). Indeed, because individual differences in vocal production anatomy directly affect the spectral acoustic structure of the emitted signal [25], [35], the presence of identity cues in mammal vocal signals is not surprising. Our results confirm the prominent role of filter-related features as cues to identity in male koalas. In contrast, source-related features were far poorer at classifying male bellows to individuals, perhaps due to their greater propensity to change according to the caller's motivational state [26], [36].

Other studies on humans and nonhuman mammals have emphasized the importance of filter-related acoustic features as cues to individual identity [2], [26], [36]–[38]. In particular, formants are likely to be a reliable source of identity cues in mammals that do not perform dynamic vocal tract modifications during call production (so that the formants are comparatively stable within individuals across calls) and in which the source energy of the call adequately highlights the caller's distinctive formant pattern (Rendall et al., 1998; Owren and Rendall 2003; Rendall, 2003). Because the spectral prominences of male koala bellow inhalation phases are relatively stable (see Figure 1b), and therefore consistent among the calls an individual makes, they are likely to be highly individually distinctive. In addition, observations of male bellow spectrograms and power spectra show a pulse-train structure that should emphasise characteristics of the supra-laryngeal filter (see Figure 1b), increasing their salience to receivers (as discussed by [39], [40], [41]). Accordingly, the pulse-train structure of male koala bellows may even have evolved to facilitate vocal recognition by highlighting a caller's distinctive formant pattern.

It is interesting to note that the average frequency spacing based on the first six spectral prominences of the inhalation phases of male bellows was 356 Hz. If these spectral peaks represent formants then using the following equation: VTL = c/2ΔF where c is the approximate speed of sound in the mammalian vocal tract (350 m/s) and ΔF is the formant frequency spacing, this predicts a vocal tract length of 49 cm. Post mortem investigations and magnetic resonance imaging scans indicate, however, that male koala supra-laryngeal vocal tract length is much shorter than this, with a resting larynx position that affords a vocal tract length of around 15 cm (Charlton & Fitch et al. Unpublished data). Further anatomical investigations show that the sterno-thyroid muscles are anchored very deep in the thoracic cavity (Charlton & Fitch et al. Unpublished data). While this may indicate that koalas have the ability to retract the larynx to the sternum during vocalizations, this would still only increase vocal tract length to around 20 cm (the distance from the incisors to the sternum: unpublished data) and hence, could not explain our observations.

Nevertheless, since the spectral prominences are not harmonically related (i.e., they are not multiple integers), are separated by over 300 Hz, and present in sections of bellows with a stable and very low F0 of around 20 Hz, they could not be harmonics of the fundamental and thus, are likely to represent supra-laryngeal resonances. Future research employing visual imaging techniques will be needed to further examine the anatomical basis for our findings. As it stands, however, cues related to vocal tract filtering appear to contribute the most to vocal identity in male koala bellows.

### Discrimination of caller identity

The playback experiments show that koalas can discriminate between the bellow vocalizations of different males. In addition, because the subject's sex did not affect their propensity to dishabituate or rehabituate (as indicated by the lack of a significant interaction between subject sex and playback type), we suggest that the identity of male koalas is important for both sexes. Whether koalas are able to vocally recognise different males or if they just discriminate between the vocalisations of different callers is less clear. Nevertheless, our demonstration that koalas react to changes in male callers indicates that vocal recognition of males could occur in reproductive contexts, and that the identity of male callers is functionally relevant to male and female koalas during the breeding season.

Previous studies investigating vocal recognition in mammals typically focussed on mother-offspring recognition [11], [12], and calls produced in the contexts of group cohesion [6], [8], [42] and alarm calling [9], [10]. Very few studies to date have considered the role of vocal recognition in sexual contexts (but see [31], [43]) and none in a marsupial species. Male koalas often bellow after agonistic interactions and in response to other bellows [20], [23]. Individually distinctive bellows could, therefore, allow males to make judgements on whether or not to escalate competitive interactions based on their previous experience with a given individual. Moreover, male koalas often remain in the same general location for several years and stable dominance hierarchies are reported to exist [20]. Thus, adult male koalas could learn to identify other males in their vicinity and individual vocal characteristics may enable the establishment of specific dominance relationships (as suggested in other seasonal breeders: [19], [44], [45]). Female koalas could also become familiarised to the bellows of high quality/dominant males that can outcompete rivals and prefer these individuals in mate choice contexts [18], [19], [43]. Indeed, vocal recognition in these contexts is especially likely to be important in this arboreal, relatively cryptic species in which other sensory modalities may be less efficient for signalling identity.

The particular features that koalas attend to when discriminating between callers is not known. However, our acoustic analysis indicated that F4–F6 were the most individually distinctive acoustic features of male bellows and consequently, these acoustic features could be important for vocal recognition. Furthermore, while the overall pattern of filter-related acoustic features is likely to be distorted by attenuation effects that will not be constant across the frequency domain (as found in elephants: [42]) koalas may even be able to discriminate between male callers based on F4–F6 alone. Future work should re-record male bellows in eucalyptus forests to assess the degradation of specific acoustic features and determine the distances over which information on caller identity could realistically be broadcast. In addition, resynthesis techniques could be used to standardise the values of F4–F6 across habituation–discrimination sequences, in order to investigate the importance of these filter-related acoustic features for vocal recognition in this species.

To conclude, male koala bellows are highly individually distinctive and filter-related features contribute the most to vocal identity. In addition, our results show that male and female koalas react to a change in the identity of male callers, indicating that caller identity is functionally relevant to both sexes during the breeding season. The precise function of signalling caller identity in this species remains a topic for future research; however, we suggest that vocal recognition may be important for males and females in the contexts of intra-sexual competition and mate choice, respectively. Furthermore, since male koala bellows are individually distinctive, the acoustic structure of these calls might be used as a bioacoustic tool for assessing local population densities. This type of information is crucial for determining the conservation status of specific populations and is difficult to obtain in koalas, because they are relatively cryptic and typically occur in low abundance [21]. However, given the high rates of male koala vocal activity during the 3–4 month breeding season [22], remote recording sensors could capture enough good quality recordings for bioacoustic techniques to be used to help distinguish between different individuals. Because koala sex ratios are fairly uniform [21] the adult population size could then be estimated by extrapolation, aiding management and conservation efforts in this iconic species.

## Materials and Methods

### Study site and animals

The koalas involved in this study were housed at Lone Pine Koala Sanctuary (LPKS), Brisbane, Australia, and the Queensland Parks and Wildlife Service Moggill Koala Hospital (MKH), Moggill, Brisbane, Australia. All the animals were individually recognizable and sexually mature (<3 years old). Tooth wear was used to confirm the adult status of the wild-sourced koalas at MKH.

### Ethical statement

This work follows the Association for the study of Animal Behaviour/Animal Behaviour Society guidelines for the use of animals in research, and was approved by the University of Queensland Animal Ethics Committee (approval number SAS/227/10).

### Recordings

Male bellows were recorded from 20 adult koalas (aged 3–15 years) at LPKS using a Sennheisser ME67 directional microphone and a Zoom H4N portable solid-state digital recorder (sampling rate: 44.1 kHz, amplitude resolution: 16 bits) at distances ranging from 1–10 meters. Recordings were transferred to an Apple Macintosh Macbook Pro computer, normalized to 100% peak amplitude and saved as WAV files (44.1 kHz sampling rate and 16 bits amplitude resolution). The overall spectral structure of each bellow was initially investigated using narrow-band spectrograms (see Figure 1: FFT method; window length 0.05 s; time step = 0.004 s; frequency step = 10 Hz; Gaussian window shape; dynamic range = 35 dB) and recordings with high levels of background noise were discarded. In addition, to limit the affect of daily variation in arousal levels, a minimum of 10 bellows from each male were used that had been recorded on at least four different days. This gave us a total of 276 bellows from 20 males for the acoustic analysis (see Table 1 for the number of bellows recorded from each subject).

### Acoustic Analyses

We selected features of male bellows that would reflect acoustic differences generated by both the source and filter for the acoustic analyses. Purpose built scripts in PRAAT DSP package version 5.0.29 (www.praat.org) were used to extract a range of source- and filter- acoustic measures, before automatically logging these variables into an output file.

#### a. Source-related measures

Because the exhalation sections of male bellows are typically characterised by deterministic chaos (episodes of non-random noise produced by chaotic vocal fold vibration) with no clear F0 or harmonic structure, we only considered the inhalation sections of male bellows for the analysis of F0-related features. The F0 contour was extracted using the ‘To pitch (ac) command’ (time step = 0.01 s; voice threshold = 0.3; silence threshold = 0.2; minimum and maximum F0 = 10 Hz and 100 Hz, respectively) integrated into a purpose-built script. This script returned the minimum, mean, and maximum F0 values in hertz. Time-varying numerical representations of the F0 contour were compared with narrow-band spectrograms, checked for any ‘octave jumps’, and played back (as a pulse train) for subjective comparison with the original recording. Any incorrect values were ‘unvoiced’ in the Pitch edit window and sections with deterministic chaos were left unmarked.

To quantify F0 stability we used a measure of F0 perturbation termed jitter. Jitter measures the cycle-to-cycle variability in F0 across the call and may contribute to vocal distinctiveness in mammals [26]. In our analysis jitter was expressed as a percentage, and to increase the validity of our measurements (and the applicability of our results) we averaged three measurement values: local, relative average perturbation, and 5-point period perturbation quotient. These jitter measurement algorithms in PRAAT use the ‘waveform-matching’ method that is less affected by additive noise and small amounts of amplitude fluctuation than single event ‘peak-picking’ and ‘zero-crossing’ methods (Titze & Liang 1993). Finally, the call duration in seconds (duration) was measured from the waveform and, because amplitude modulation contributes to vocal recognition and distinctiveness in other animals [43], [46], [47], we also extracted the intensity contour of each entire bellow (using the sound: To Intensity command in PRAAT, minimum pitch: 10 Hz; time step auto) to measure the mean variation in amplitude per second in dB (Ampvar).

#### b. Filter-related measures

Without a more complete knowledge of the functional anatomy of the koala vocal tract it was difficult to make *a priori* predictions about the number of vocal tract resonances to expect in a given frequency range. However, using narrow-band spectrograms we identified regions encompassing several harmonics that may represent vocal tract resonances in the inhalation phases of bellows (see Figure 1b). To exclude the possibility that we were measuring energy concentrations that could still remain in sections of deterministic chaos (called ‘pseudo-formants’: [48]), we only considered the inhalation sections of male bellows with a clear pulse-train structure and stable spectral prominences for the analysis of filter-related features.

The frequency values (Hz) of the first six spectral prominences of the inhalation phases were measured using Linear Predictive Coding (LPC; ‘To Formants (Burg)’ command in PRAAT. To check if PRAAT was accurately tracking these frequency components we compared the outputs with visual inspections of each call's spectrogram and power spectrum (using cepstral smoothing: 200 Hz) before using the following analysis parameters: time step: 0.01 seconds; window analysis: 0.03 seconds; maximum formant value: 2300 Hz; maximum number of formants: 6; pre-emphasis: 50 Hz. The average frequency spacing of the spectral prominences (ΔF) achieved during each vocalisation was then estimated using the regression method of Reby and McComb [49], in which the observed frequency values (F1–F6) are plotted against those that would be expected if the vocal tract was a straight uniform tube, closed at one end and open at the other.

### Playback Experiments

The playback experiments were conducted at MKH during the 2010 breeding season on 12 adult koalas (6 male and 6 female). All the subjects were separately housed in cubicles measuring approximately 2.0×3.0×3.0 m.

#### a) Playback stimuli

To construct the playback sequences we selected bellows of comparable duration (22.1±3.9 seconds) from six adult males that were aged between 3–15 years (mean = 9 years). These males were recorded by BC at Lone Pine Koala Sanctuary using a Sennheisser ME67 directional microphone and a Zoom H4N portable solid-state digital recorder (sampling rate: 44.1 kHz, amplitude resolution: 16 bits) and hence, were unfamiliar to the current residents at MKH. The mean relative intensity values for all the playback stimuli were standardized to 60 dB using the ‘Scale intensity’ command in Praat. A preliminary analysis on the male bellows used to investigate inter-individual differences in male vocal characteristics indicated that age was not correlated to any of the acoustic features of bellows we measured (*N* = 20; *P* all >0.4). Consequently, no attempt was made to match exemplars according to age. However, to limit the possibility of dishabituation occurring due to size-related acoustic differences the male exemplars were paired up based on their head length (measured from the tip of the nose to the occipital ridge, and ranging from 13.4–14.5 mm).

The habituation-discrimination playback sequences consisted of seven male bellows separated by 40 seconds (constituting a realistic rate of delivery for these vocalizations: [23]). In each sequence the first five bellows comprising the habituation phase (H1–H5) and the 7th bellow, the rehabituation stimulus (RH), originated from the same male exemplar. The 6th bellow of the playback sequence, the dishabituation stimulus (DH), originated from a different male to that of the habituation phase. In this way we aimed to provoke habituation to bellows H1–H5 with discrimination inferred if the level of response significantly increased to the dishabituation stimulus (DH) and then fell back again after the rehabituation stimulus (RH). If koalas dishabituate because of a particular difference between H5 and DH then their response levels should remain high to RH, because it is again equally dissimilar to DH. In addition, we used different original calls for each of the habituation stimuli to ensure that subjects become habituated to the individual content of different calls and not to the repetition of a single call [8], [30], [31]. The habituation phase comprised five playbacks, and to ensure a symmetrical experimental design each male was used alternately to habituate and dishabituate subjects, giving us six unique sequences from the three male pairs.

#### (b) Playback execution

The playbacks were initiated when subjects were settled and their attention was directed away from the speaker position. This ensured a standardised context at the onset of each experiment. We played back male bellows using an Anchor Audio Explorer Pro 6000 loudspeaker at sound pressure levels equivalent to that of naturally bellowing males of 65 dB SPL peak (determined using a Radio Shack Sound Level Meter, set for C-weighted fast response and measured 1 meter from the source), and balanced the presentation of each of the six unique playback sequences across the 12 subjects. The subject's behaviour was videotaped during the experimental period using a Canon LEGRIA FS20 digital video camcorder mounted on a tripod.

### Behavioural analysis

To quantify the strength of each subject's response during the experimental period the videotapes were analysed frame-by-frame (using Gamebreaker v7 digital video analysis system for Mac OS 10.5, SportsTec Sydney) and the duration of the first look given towards the speaker was measured. The subject was deemed to be looking towards the playback source when their head was oriented towards the speaker having previously faced away. Sometimes subjects were already looking towards the speaker when one of the bellows of a sequence was broadcast. In these cases, looking behaviour began at the onset of the playback. A movement leading to the subject looking away from the speaker position or the subject closing their eyes was considered to be the end of the behaviour.

### Statistical analyses

All statistical analyses were conducted using SPSS version 16 and significance levels were set at p = 0.05. In order to evaluate individual differences in the acoustic structure of male bellows we performed a Discriminant Function Analysis (DFA) to classify the calls, with subject identity as the group identifier and the acoustic measures as discriminant variables. For each classification, both the reclassification and the more conservative leave-one-out cross-validation procedure were applied. In addition, to assess the stability of vocal individuality, we ran a second DFA to classify calls recorded on the last day for each individual using models trained with calls recorded on the other previous days (‘hold-out-sample’ method: [50]). Because of uneven subject participation in the dataset the percentage correct classification expected due to chance was calculated according to the group sizes (see Table 1). The statistical significance of correct classification of bellows to each male and across all subjects was obtained using the Chi square statistic and two-tailed probability values were used.

For the analysis of the playback data we used a two-way mixed general linear model. The within-subjects factor ‘playback type’ comprised three levels: H5, DH and RH, and a difference contrast was used to detect significant differences in the mean first look duration between H5 and DH, to test for dishabituation, and DH and RH, to test for rehabituation. To investigate any sex differences in overall looking response we entered the subject's sex as a between-subjects factor. We also tested for a possible interaction between playback type and sex, to detect whether the subject's sex had an affect on their propensity to dishabituate or to rehabituate. Before statistical testing the data was log_10_ transformed to normalize the distribution for parametric tests. To retain zeros in the dataset a value of one was added to each data point before transformation. The data were also tested for homogeneity of variance (Levene's test) and sphericity (Mauchly's test) and both assumptions were met (P>0.05). Finally, one-tailed probability values are quoted for the analysis of the playback data because a decrease in response would be regarded as further habituation in our experiment and therefore equivalent, using this experimental paradigm, to no perception of the change in test stimulus [51], [52]. Notwithstanding this, all results would remain significant with a two-tailed approach.

## Supporting Information

Movie S1 **A video recording of a bellowing male koala (Andy) at LPKS (MOV).**
(MOV)
